# Pulmonary Vascular Responses to Chronic Intermittent Hypoxia in a Guinea Pig Model of Obstructive Sleep Apnea

**DOI:** 10.3390/ijms25137484

**Published:** 2024-07-08

**Authors:** Elena Olea, Esther Valverde-Pérez, Inmaculada Docio, Jesus Prieto-Lloret, Philip I. Aaronson, Asunción Rocher

**Affiliations:** 1 Departamento de Enfermería, Facultad de Enfermería Universidad de Valladolid, 47005 Valladolid, Spain; elena.olea@uva.es; 2 Unidad de Excelencia Instituto de Biomedicina y Genética Molecular (IBGM), Universidad de Valladolid-CSIC, 47005 Valladolid, Spain; esther.valverde.perez@uva.es (E.V.-P.);; 3 Departamento de Bioquímica y Biología Molecular y Fisiología, Facultad de Medicina, Universidad de Valladolid, 47005 Valladolid, Spain; 4 Department of Inflammation Biology, School of Immunology and Microbial Sciences, Faculty of Life Sciences and Medicine, King’s College London, London SE1 9RT, UK

**Keywords:** systemic and pulmonary hypertension, intermittent hypoxia, hypoxic pulmonary vasoconstriction, carotid body, autonomic nervous system, obstructive sleep apnea, endothelial function, vessel remodeling, guinea pig

## Abstract

Experimental evidence suggests that chronic intermittent hypoxia (CIH), a major hallmark of obstructive sleep apnea (OSA), boosts carotid body (CB) responsiveness, thereby causing increased sympathetic activity, arterial and pulmonary hypertension, and cardiovascular disease. An enhanced circulatory chemoreflex, oxidative stress, and NO signaling appear to play important roles in these responses to CIH in rodents. Since the guinea pig has a hypofunctional CB (i.e., it is a natural CB knockout), in this study we used it as a model to investigate the CB dependence of the effects of CIH on pulmonary vascular responses, including those mediated by NO, by comparing them with those previously described in the rat. We have analyzed pulmonary artery pressure (PAP), the hypoxic pulmonary vasoconstriction (HPV) response, endothelial function both in vivo and in vitro, and vascular remodeling (intima–media thickness, collagen fiber content, and vessel lumen area). We demonstrate that 30 days of the exposure of guinea pigs to CIH (FiO_2_, 5% for 40 s, 30 cycles/h) induces pulmonary artery remodeling but does not alter endothelial function or the contractile response to phenylephrine (PE) in these arteries. In contrast, CIH exposure increased the systemic arterial pressure and enhanced the contractile response to PE while decreasing endothelium-dependent vasorelaxation to carbachol in the aorta without causing its remodeling. We conclude that since all of these effects are independent of CB sensitization, there must be other oxygen sensors, beyond the CB, with the capacity to alter the autonomic control of the heart and vascular function and structure in CIH.

## 1. Introduction

Obstructive sleep apnea (OSA), characterized by the repeated occurrence of apneas and hypopneas during sleep, is one of the most common chronic diseases, affecting up to nearly one billion people worldwide [1]. This breathing disorder is strongly associated with cardiovascular disease, as sleep apnea is widely recognized as a major independent risk factor for hypertension, atherogenesis, coronary heart disease, cardiac arrhythmias, heart failure, and stroke [2,3].

Among the pathophysiological mechanisms of OSA, intermittent hypoxia (IH) caused by repetitive hypoxia-reoxygenation cycles is thought to be the key intermediary mechanism leading to cardiovascular morbidity and mortality [4,5,6]. However, the mechanisms underlying the association between OSA and cardiovascular disease are not fully understood. In this regard, several animal models have been developed to study the effects of chronic intermittent hypoxia (CIH) on the cardiovascular system. Numerous studies in rodents support the hypothesis that CIH is responsible for the increased arterial blood pressure, structural vascular remodeling, altered vascular reactivity, and progression of atherosclerosis observed in OSA.

There is considerable evidence that, when exposed to CIH, the carotid body (CB), the major peripheral oxygen sensor, becomes pathologically hyperactive, leading to the chronic activation of chemoreflexes, which increases the afferent traffic to the brainstem, respiratory plasticity, and sympathetic outflow responsible for the increased blood pressure [7,8]. CB denervation or ablation blocks CIH-induced changes in ventilation and systemic hypertension, demonstrating that chemoreflexes are essential not only for the development of respiratory plasticity but also for the cardiovascular events. In addition, CIH promotes oxidative stress and inflammation in a CB-dependent and CB-independent manner [9], which may contribute to the onset of vascular dysfunction and the development of sustained hypertension in CIH-exposed animals and in patients with OSA [10,11,12].

It has also been suggested that OSA may contribute to the development of pulmonary hypertension (PH) and right ventricular failure through pulmonary vasoconstriction secondary to hypoxia. In addition, CIH has been shown to cause pulmonary vascular remodeling associated with thickening of the medial layer, an effect shown to be due to the increased expression of proteins mediating store-operated Ca^2+^ entry in pulmonary artery smooth muscle cells [13]. However, other studies suggest that CIH is not sufficient to cause sustained PH, as occurs with sustained alveolar hypoxia, for example in patients with chronic obstructive pulmonary disease (COPD) and in animal models with chronic sustained hypoxia (CSH). A recent study in rats by our group [14] showed that CIH, like CSH, produces an elevation in pulmonary arterial pressure (PAP), although one smaller than that caused by CSH, in addition to different effects on ventricular hypertrophy and the contractile properties of pulmonary arteries (PAs). The development of left rather than right ventricular hypertrophy may be secondary to the development of systemic hypertension in CIH rats. Regarding contractile properties, our results showed that CIH exposure slightly increased phenylephrine PE-induced contraction but had no effect on hypoxic pulmonary vasoconstriction (HPV) or endothelium-dependent relaxation in rat PA [14]. It has also been reported that mice exposed to CIH had lungs with significantly more muscularized small pulmonary vessels (positive staining for myosin) compared to normoxic mice [15].

Because we are interested in evaluating the involvement of the CB in the cardiovascular and pulmonary effects of CIH, we recently developed a model of CIH in the guinea pig, an animal with a hypoxia-insensitive CB that can be considered a natural CB knockout. In a previous work [16], we demonstrated that CIH in the guinea pig induced sympathetic hyperactivity with increased circulating catecholamines that promoted cardiovascular adaptations such as increased heart rate and mean arterial systemic pressure, without significant changes in the hypoxic ventilatory response, i.e., effects that were apparently not mediated by CB chemoreflex activation.

Here, we hypothesize that prolonged exposure to CIH in the guinea pig may alter pulmonary hemodynamics and cause PH, as in rats [14], if these effects are CB-independent. To date, no study has investigated pulmonary vascular function using the same experimental model of CIH in the guinea pig. Therefore, the present study was designed to characterize the functional consequences of chronic exposure to intermittent hypoxia on PAP, the HPV response, endothelial function in both in vivo and in vitro (arterial rings) vessels, and vascular remodeling by proliferating PA smooth muscle cells (PASMCs; intima–media thickness; IMT and vessel lumen area). As information about the response of the systemic vasculature to hypoxia in this species is also lacking, as well as confirming its in vivo effects on hemodynamics we additionally explored the effects of CIH on the structure and regulation of vascular contractility in the aorta and carotid arteries and on the expression of endothelium-derived pulmonary vasodilators and endogenous inhibitors of the nitric oxide (NO) system. Our results demonstrate that CIH exerts multiple effects on the structure and contractile function of the pulmonary and systemic vasculatures that do not require a CB response.

## 2. Results

### 2.1. Animal’s General Status, Body Weight, and Hematocrit

All the animals completed the study in good condition. The body weight, arterial gasometry, and hematocrit of both the CIH and C groups after the 30-day CIH period are shown in Table 1. Body weight was significantly lower in the CIH than in the C group (*p* < 0.01). CIH caused a significant decrease in weight gain from the first week of exposure. As a result, at the end of the study (day 30), body weight in the experimental group was significantly lower than that in the control group. Hematocrit was not significantly different in the hypoxic compared to the normoxic group, although EPO increased in CIH animals (*p* < 0.05). These effects of CIH were the same as those observed by Docio et al. [16].

### 2.2. Pulmonary Hemodynamics and Right Ventricle Hypertrophy

**Figure 1:**
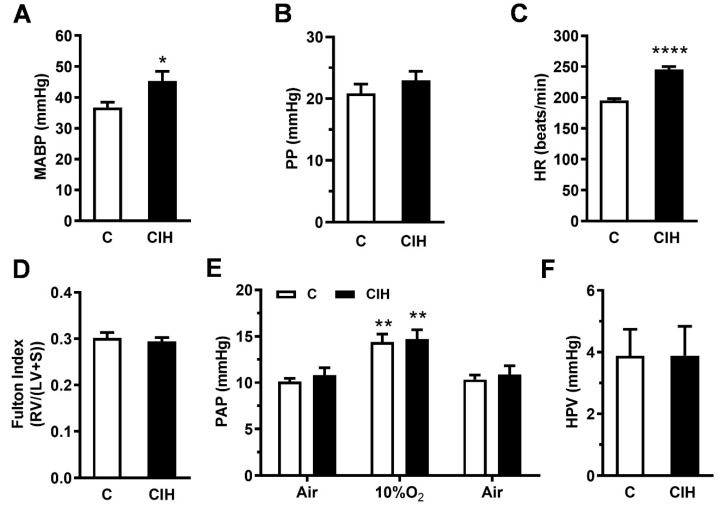
(**A**) Mean systemic arterial blood pressure (mmHg), (**B**) pulse pressure (mmHg), (**C**) heart rate (beats/minute), (**D**) Fulton index (right ventricle/left ventricle + septum), (**E**) pulmonary arterial pressure (mmHg), and (**F**) hypoxic pulmonary vasoconstriction (mmHg) in control (**C**) and CIH guinea pigs in hypoxia (10% O_2_). Data are expressed as mean ± standard error (SEM) of N = 7 animals/group. Unpaired *t*-test (**A**–**D**,**F**) and two way ANOVA (**E**). (* *p* < 0.05; ** *p* < 0.01; **** *p* < 0.0001).

To assess the impact of CIH exposure on systemic and pulmonary hemodynamics, direct measurements of arterial pressure in the carotid and main PA were obtained by catheterization in guinea pigs ventilated with 21% O_2_, except during the measurements shown in Figure 1F. When compared with the control group, CIH exposure increased the mean systemic arterial blood pressure (MABP) from 37 ± 2 mmHg to 45 ± 3 mmHg (*p* < 0.05; Figure 1A) as both systolic and diastolic pressures were increased in CIH-exposed guinea pigs, although the pulse pressure (PP) did not change significantly (Figure 1B). However, a significantly increased heart rate (25%) was seen in CIH guinea pigs compared to control animals (245 ± 5 vs. 195 ± 3 beats/m; **** *p* < 0.0001; Figure 1C).

To determine the impact of blood pressure alterations on ventricular cardiac mass, the Fulton index was calculated (Figure 1D). CIH-exposed guinea pigs did not show RV hypertrophy, as the RV/LV + S ratio was not different from the C group.

Finally, baseline normoxic PA pressure was found not to be significantly different in CIH compared to controls (10.8 ± 0.8 vs. 10.1 ± 0.4 mmHg; Figure 1E). PAP similarly increased to ~15 mmHg during acute hypoxic challenge (10% O_2_ during 3 min) in both the C and CIH groups, reflecting a completely functional HPV in this experimental model (Figure 1E). In summary, in the guinea pig, CIH increases MABP and HR but it does not change the Fulton index, PAP, or HPV.

### 2.3. Vascular Contractility and Endothelial Function

The mechanical properties and reactivity of the pulmonary, aorta, and carotid arteries were assessed. Table 2 shows the vascular responses to 80 mM KCl in the aorta, carotid, and PA. The maximal contraction induced by KCl in aorta was lower in animals exposed to CIH (but not significantly), whereas in the pulmonary and carotid arteries it was similar to that in the C group.

**Figure 2:**
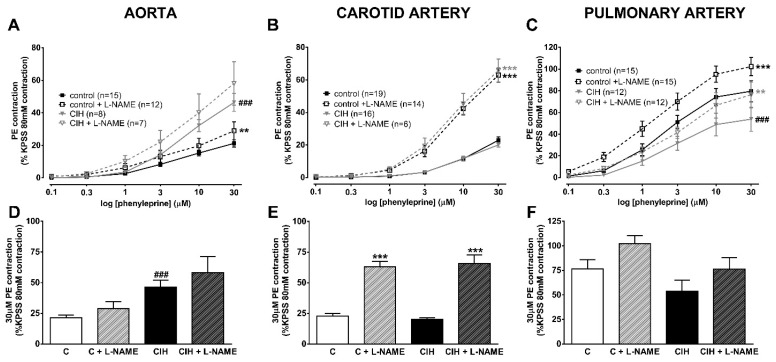
Vascular contractile responses to PE in aorta (panel **A**), carotid artery (panel **B**), and pulmonary artery (panel **C**) with or without 0.1 mM L-NAME in control and CIH guinea pigs. Data are expressed as mean ± standard error (SEM). Two-way ANOVA with Sidak’s multiple comparison test; ** *p* < 0.01; *** *p* < 0.001, L-NAME vs. its control. Vascular contractile responses to 30 µM PE in aorta (panel **D**), carotid artery (panel **E**), and pulmonary artery (panel **F**) with or without 0.1 mM L-NAME in C and CIH guinea pigs. Data are expressed as mean ± standard error (SEM), n = 7–15 for aorta; n = 6–19 for carotid artery; n = 12–16 for pulmonary artery, where n are the replicates of N = 6 animals/group. Unpaired *t*-test *** *p* < 0.001 C or CIH vs. C + L-NAME or CIH + L-NAME; ^###^ *p* < 0.001 C vs. CIH.

Figure 2 shows the vascular contractile responses to cumulative doses of PE (0.1–30 µM) in the aorta (panel A), carotid (panel B), and pulmonary artery (panel C) and the effect of the NOS inhibitor L-NAME. PE is a synthetic alpha 1-adrenoreceptor agonist with moderate potency as a vasoconstrictor. Like the receptors for most arterial vasoconstrictors, alpha1 adrenoceptors are thought to primarily cause contraction by stimulating the IP_3_/DAG second messenger system, resulting both in a rise in the intracellular Ca^2+^ concentration and Ca^2+^ sensitization.

PE-induced contraction was higher at all concentrations in the aorta of CIH compared to C guinea pigs in the presence or absence of NO (L-NAME pretreatment). In contrast, the response to PE was virtually identical at all doses in carotid arteries from C and CIH animals in both the presence and absence of L-NAME. The low contractile values reached in both systemic arteries (~20% KPSS) are striking.

In PA, the PE-induced contraction was relatively larger than in the systemic arteries (>50% KPSS response) but was lower at all concentrations in the CIH than in the C group. The inhibition of eNOS similarly enhanced contraction in both groups (dashed line), ruling out a higher production of NO in CIH guinea pigs as the cause of the lower contractibility than in C guinea pigs.

Figure 2 shows that L-NAME almost tripled the 30 µM PE contraction in the carotid artery (panel E) of the CIH compared with C guinea pigs. In contrast, the response to PE was not significantly different in the presence or absence of L-NAME in the aorta (panel D) and PA (panel F) from either group. These data suggest that alpha1-receptor activation evokes NO release, which greatly limits its ability to vasoconstrict the carotid artery.

**Figure 3:**
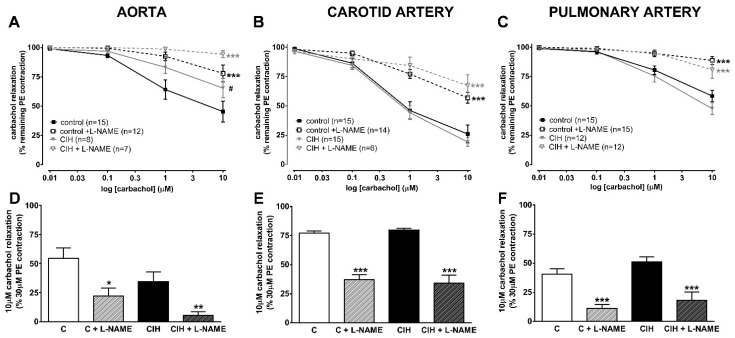
Vascular relaxation responses to carbachol in aorta (panel **A**), carotid artery (panel **B**), and pulmonary artery (panel **C**), with or without 0.1 mM L-NAME, in C and CIH guinea pigs. Data are expressed as mean ± standard error (SEM). Two-way ANOVA with Sidak’s multiple comparison test; *** *p* < 0.001 (C or CIH vs. C + L-NAME or CIH + L-NAME); ^#^ *p* < 0.05 C vs. CIH. Vascular relaxation to 10 µM carbachol in aorta (panel **D**), carotid artery (panel **E**), and pulmonary artery (panel **F**) with or without 0.1 mM L-NAME. Data are expressed as mean ± standard error (SEM), n = 7–15 for aorta; n = 6–19 for carotid artery; and n = 12–16 for pulmonary artery, where n are the replicates of N = 6 animals/group. Unpaired *t*-test (* *p* < 0.05; ** *p* < 0.01; *** *p* < 0.001) (C or CIH vs. C + L-NAME or CIH + L-NAME).

Figure 3 shows the changes in arterial tension caused by cumulative concentrations of carbachol (10 µM–10 µM) on top of a maximal PE contraction. This protocol was used to test endothelial functionality because carbachol releases NO from the endothelial layer of the artery. In the aorta (panel A), endothelium-dependent vasorelaxation was significantly blunted at each concentration in CIH animals compared to controls (*p* < 0.05), indicating the presence of endothelial dysfunction in this artery. Concomitant treatment with L-NAME almost completely abolished the endothelium-dependent vasodilator response in the CIH group and strongly depressed it in the C group. In contrast, no differences were observed in endothelium-dependent vasorelaxation in carotid (panel B) and PA (panel C) in the CIH group compared to the C group (50% and 75% reduction, respectively). The effect of carbachol was completely reversed by L-NAME in the pulmonary artery, whereas L-NAME-induced reversal was only partial (~50%) in the carotid artery. In the PA from both groups, the response to carbachol with or without L-NAME was very similar, indicating a lack of endothelial dysfunction in CIH.

Figure 3D–F shows that the effect of L-NAME on 10 µM carbachol relaxation was greater in the aorta (85% vs. 60% reversal; Figure 3D) but similar in the PA (Figure 3F) of the CIH group. In the carotid artery (Figure 3E), the reversal of the L-NAME effect was only partial (~50%) but was similar in the two groups.

### 2.4. Morphometric and Histological Assessments of Vascular Remodeling

**Figure 4:**
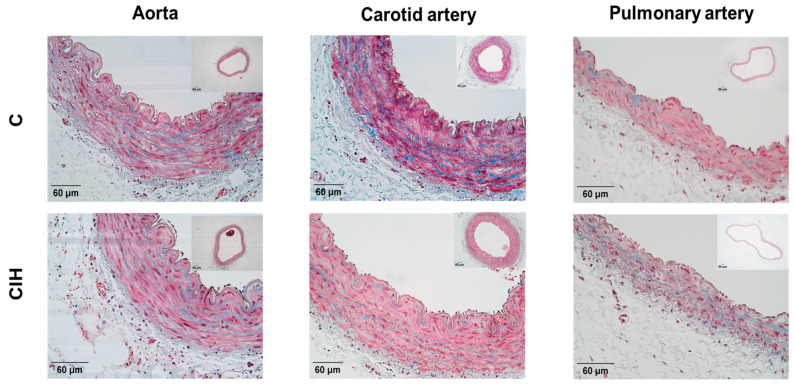
Representative images of the aorta (**left**), carotid artery (**middle**), and PA (**right**) stained with Masson’s trichrome for C (**top**) and CIH (**bottom**) groups. Scale bars: 60 μm.

To evaluate the morphology of the aorta, carotid artery, and PA from C and CIH guinea pigs, Masson’s trichrome staining, a standard way of looking at vascular morphology, was performed on 5 µm thick arterial sections from a total of six C and six CIH guinea pigs. Figure 4 shows sections of aorta (mild thoracic aorta), common carotid artery, and intralobar PA from control (top) and CIH (bottom) guinea pigs taken with the 4× or 10× objective with a magnified area taken with the 20× objective. This staining allows the visualization of the muscle layer (pink) and the collagen (blue green) present In the arteries to analyze the morphology of the vessels. The collagen content was quantified as a percentage of total IMT area. The insets in each figure correspond to the complete section of each artery in which the area of the lumen has been measured.

**Figure 5:**
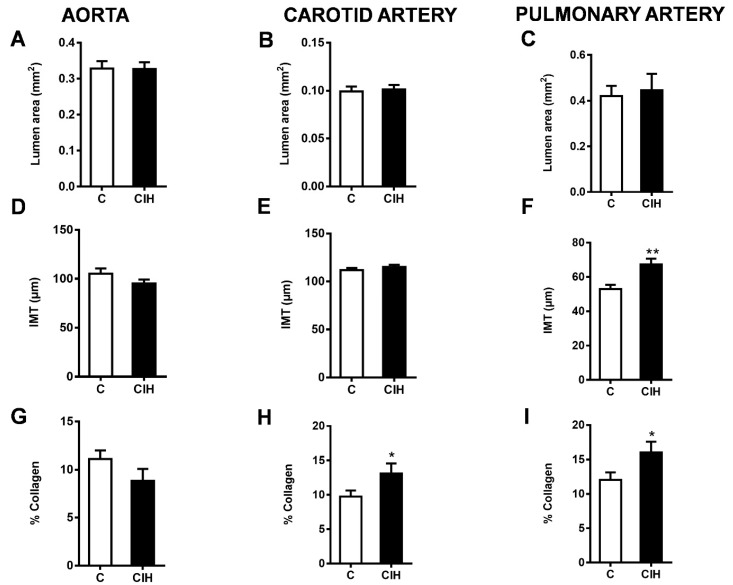
Morphometric and histologic evaluation in the aorta (left), carotid artery (middle), and PA (right). (**A**–**C**) Lumen area for each artery. (**D**–**F**) Intima–media thickness (IMT) for each artery. (**G**–**I**) Collagen deposition expressed as a percentage of the IMT area for each artery. Data are expressed as mean ± standard error (SEM) of quintuplicate measurements (n = 30 replicates) from N = 6 animals. (Unpaired *t*-test * *p* < 0.05; ** *p* <0.01).

Figure 5 shows the morphometric and histological analysis for each artery in both conditions. The luminal area of the aorta (Figure 5A), carotid artery (Figure 5B), and intralobar PA (Figure 5C) was not modified by exposure to CIH in guinea pigs. Although intima media thickness (IMT) was not altered in the aorta (Figure 5D) and carotid artery (Figure 5E), it was significantly increased in the PA (67.4 ± 3.4 µm vs. 52.9 ± 2.5 µm in C; *p* < 0.01), as shown in Figure 5E. A significant increase in the % collagen occurs in the carotid artery, as shown in Figure 5H (13.1 ± 1.5% vs. 9.7 ± 0.9% in C; *p* < 0.05), and in the PA, as shown in Figure 5I (16.0 ± 1.6 vs. 12.0 ± 1.1 in C; *p* < 0.05), suggesting an induction of collagen synthesis during CIH exposure. No significant changes were observed in the aorta (Figure 5G).

### 2.5. NO Bioavailability: Plasma Nitrites and Nitrates and L-arginine and Its Metabolites

**Figure 6:**
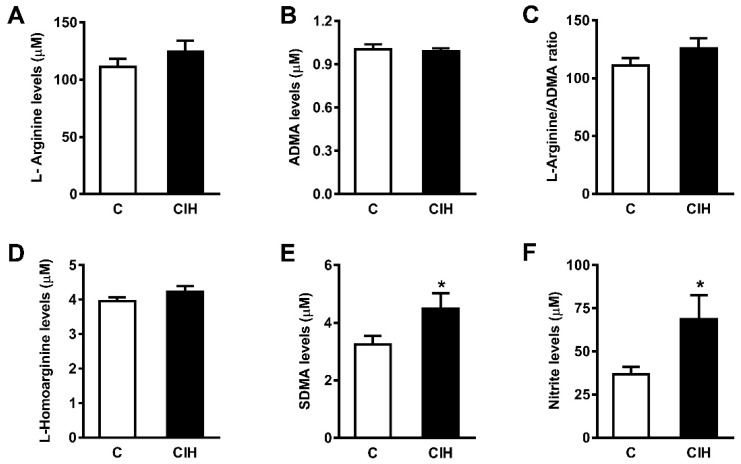
(**A**) Plasma levels of L-Arginine (µM) in control and CIH guinea pigs. (**B**) Asymmetric dimethylarginine (ADMA). (**C**) L-Arginine/ADMA ratio. (**D**) L-Homoarginine (µM). (**E**) Symmetric dimethylarginine (SDMA). (**F**) Plasma Nitrites (µM) in control and CIH guinea pigs. Data are expressed as mean ± standard error (SEM) of N = 8 animals. Unpaired *t*-test (* *p* < 0.05).

Circulating vasoactive factors were studied to determine the status of the substances most likely to affect the endothelial function in guinea pig vessels and the effect of CIH on them. First, the bioavailability of NO was assessed by quantifying nitrites and nitrates, both metabolites of NO, and L-arginine, a natural precursor of NO, and its metabolites asymmetric dimethylarginine (ADMA) and symmetric dimethylarginine (SDMA). As shown in Figure 6A, the plasma levels of nitrites and nitrates were doubled in animals exposed to CIH (69 ± 14 µM vs. 37 ± 4 µM in C; *p* < 0.05). L-Arg (125.9 ± 9.1 vs. 111.4 ± 6.7) and SDMA (4.5 ± 0.4 vs. 3.2 ± 0.2) increased in the CIH group, whereas ADMA and L-homoarginine were similar in both groups.

### 2.6. Plasma Endothelin-1 (ET-1), Angotensin II (ANG II), Atrial Natriuretic Peptide (ANP), Vascular Endothelial Growth Factor (VEGF), and Catecholamines

Table 3 shows that there was no effect of CIH on the plasma levels of ET-1 or ANG II. ANP and VEGF were also not altered by CIH exposure. In contrast, significant differences were observed in plasma catecholamines. Norepinephrine (NE) was significantly higher in the CIH guinea pigs than in the controls (*p* < 0.05). Plasma epinephrine (E) was also significantly higher in CIH guinea pigs (*p* < 0.05).

### 2.7. Aconitase: Fumarase Activity Ratio and Nuclear Factor Kappa B (NF-kB)

Neither the ratio of aconitase to fumarase (0.33 ± 0.05 vs. 0.30 ± 0.04 in the C group), an index of mitochondrial oxidative stress, nor NF-kB (1.02 ± 0.09 vs. 1.01 ± 0.06 mU/mL in C group), an index of inflammation, changed in the livers of guinea pigs exposed to CIH.

## 3. Discussion

This study represents the first investigation of pulmonary vascular function in guinea pigs exposed to chronic intermittent hypoxia. Intermittent hypoxia is considered the main feature of OSA associated with respiratory events that could affect sympathetic tone and arterial pressure [7,17], vascular structural remodeling and function [18,19,20], and atherosclerosis progression [21,22].

In the 1990s, Fletcher and others proposed that CIH-induced CB hyperactivity may be the driver of many of the effects that lead to hypertension and cardiovascular events in animals exposed to CIH [7,10,23,24,25] and in patients with OSA [8,26]. CB denervation or ablation blunts CIH-induced sympathetic nervous system hyperactivity and systemic hypertension [11,27]. However, it has been questioned whether an intact CB is essential to produce these effects [28]. There is evidence that neurons of the rostral ventrolateral medulla (RVL), which controls spinal vasomotor neurons, are excited by systemic hypoxia in CB-denervated rats, whereas RVL respiratory neurons are inhibited, demonstrating that the CB per se is not obligatory for the hypoxic recruitment of sympathetic neurons [29]. Hypoxia can excite putative C1 presympathetic neurons though a P2Y1 receptor-dependent mechanism [30]. There is also evidence for the direct effects of CIH on respiratory neurons in the medulla oblongata and altered respiratory autonomic coupling with implications for cardiovascular control [31].

In this context, we developed a model of CIH in the guinea pig as an experimental paradigm of OSA (FiO_2_ 5%, 8 h/day, 30 days), with the unique feature that this hypoxia-adapted rodent has a hypofunctional CB. The guinea pig CB lacks O_2_-sensitive K^+^ channels and therefore exposure to hypoxia does not induce glomus cell depolarization and a neurosecretory response [32], which is essential for chemoafferent signaling from the CB. In summary, the guinea pig can be considered a natural CB knockout species.

As expected, CIH exposure did not potentiate CB responses in the guinea pig OSA model, in contrast to the robust responses induced in rats. However, CIH was associated with sympathetic hyperactivity [16] and it promoted cardiovascular adjustments by increasing heart rate and mean arterial blood pressure without causing ventricular hypertrophy. Thus, CIH did not sensitize the CB chemoreceptor response to hypoxia in guinea pigs but promoted CB-independent cardiovascular adjustments [16]. The aim of the present study was to extend this preliminary cardiovascular study by characterizing the functional consequences of CIH exposure on the structure and responsiveness of the guinea pig vasculature. Therefore, aorta, carotid, and pulmonary artery vasoreactivity was assessed by wire myograph, and vessel remodeling by tissue staining; MABP and PAP were also recorded in vivo. To our knowledge, this is the first study to evaluate the consequences of CIH exposure on the function of guinea pig arteries from the systemic and pulmonary circulations. Our results show that guinea pigs developed hypertension and PA remodeling after four weeks of exposure to CIH. In contrast, CIH did not alter PAP, HPV, or the Fulton index during this period, nor did it induce endothelial dysfunction in PA.

These results agree with other studies in rats suggesting that CIH can cause a moderate (10–16 mmHg) increase in MABP [7,24,33]. In contrast, other authors have observed no change in MABP after 14 days [20] or even 70 days of CIH [34] in rats. Lucking et al. [28] also found that arterial blood pressure was unaffected by exposure to CIH in guinea pigs, although a β-adrenoceptor-dependent tachycardia was evident compared with controls. A direct comparison between our data and those of other studies in rats and guinea pigs is difficult because of the differences in the species and CIH protocols used, such as the severity of hypoxia, the number and duration of hypoxic cycles, the methods used to measure MABP, and the lack of studies in guinea pigs. However, it is striking that the positive results were similar in both species, demonstrating that CB involvement is not strictly necessary to induce either an increase in MABP or tachycardia, although they do not rule out the possibility that it may potentiate them.

Changes in the levels of several mediators have been suggested to contribute to OSA-associated cardiovascular morbidity. OSA patients exhibit a decreased response to NO and an increased activation of the sympathetic, angiotensin II, and endothelin systems [35,36,37]. We therefore investigated the effect of CIH on the vasorelaxation induced by carbachol, a cholinergic agonist that promotes endothelial NO release, and the contraction induced by PE, an adrenergic agonist that mimics sympathetic stimulation, in our model of CIH guinea pig. Furthermore, we measured the plasma endothelin-1, plasma NE and E, and angiotensin II levels.

Interestingly, CIH produced a different pattern of functional and structural effects in each of the three arteries we investigated. CIH had no effect on the responses to high K^+^, PE or carbachol in the carotid artery. The carbachol response was greatly depressed by L-NAME in these arteries, as it was in the aorta and PA, indicating that it was mainly mediated by NO release in all three vessels. It is notable that NO release also appeared to be strongly suppressing the PE contraction, as evidenced by the marked increase in the PE contraction in the presence of L-NAME. The enhancement of vasoconstriction by eNOS blockade is thought to be associated with the presence of myoendothelial gap junctions, which provide a pathway by which the increase in smooth muscle cell [Ca^2+^]_i_ evoked by vasoconstrictors can also raise [Ca^2+^]_i_ in the endothelium, causing a stimulation of eNOS that curbs the contraction [38]. The observation that L-NAME similarly enhanced the PE contraction in carotid arteries from the control and CIH groups suggests that this mechanism of NO release was also not altered by exposure to intermittent hypoxia.

CIH also had no effect on the contractions evoked by high K^+^ or hypoxia or on carbachol-induced vasorelaxation in PA. However, it decreased the amplitude of the PE contraction by ~30%. In line with the lack of effect of CIH on endothelium-dependent vasorelaxation, the depression of the PE response persisted in the presence of L-NAME, again suggesting that CIH does not affect endothelial NO production in these arteries. We have previously shown that the exposure of rats to an identical CIH protocol similarly did not alter either HPV or carbachol-induced vasorelaxation in PA, although the response to PE was modestly increased [14]. Similarly, CIH has been shown to increase the contractile response to high K^+^ and ET-1 in rats [39,40]. It is possible that the increased pulmonary artery collagen content induced by CIH could explain the decreased responsiveness of these arteries to PE in guinea pig [41]. Interestingly, CIH also caused a small but significant increase in PAP in rats, whereas in the present study it had no effect on PAP in guinea pigs. This suggests that a fully functional CB may be required for CIH to induce pulmonary hypertension.

In the aorta, as in the other two arteries, CIH had no significant effect on the high K^+^ contraction. However, the constriction elicited by PE was increased and carbachol-induced vasorelaxation was attenuated. Interestingly, L-NAME had little effect on the amplitude of the PE contraction in the aorta, consistent with the lack of myoendothelial gap junctions reported in this artery [42]. These results suggest that CIH is likely to enhance aortic vascular tone in vivo, especially in the face of the increased sympathetic drive that occurs under these conditions.

These data are not consistent with previous findings in rats showing that exposure to CIH (FiO_2_ 5%, 8 h/day for 35 days) did not alter the endothelium-dependent relaxation elicited by acetylcholine in the aorta [43]. Furthermore, whereas it was found that aorta from control and CIH rats had similar contractile responses to KCl, norepinephrine, angiotensin II, and endothelin-1, the contractile response to endothelin-1 was weakly but significantly increased in carotid artery from CIH rats in the same study [43]. A heterogeneous distribution in the receptor density of vasoactive agents in different vascular beds, such as endothelin-1 [44], has been proposed to account for the changes in the response to different vasoactive agents [43]. Along the same lines, a heterogeneous distribution of adrenoceptors in arteries has also been reported and could explain the different effect on aorta and carotid artery that we found in the guinea pig after CIH exposure [45].

Intermittent hypoxia also had different effects on the structure of each artery. Despite altering contractile function in the aorta, CIH did not affect IMT or the collagen content in this artery. In the pulmonary artery, CIH increased the accumulation of collagen fiber and also the IMT, whereas in the carotid artery only the collagen fiber content was enhanced.

Patients with OSA have been shown to have increased carotid artery IMT [46]. IMT remodeling is considered an early predisposing event in atherosclerosis and plaque formation and is associated with increased cardiovascular risk [47]. Our results showed that CIH promotes vascular remodeling with increased IMT in the pulmonary artery but not in the aorta and carotid artery of guinea pigs. Vascular remodeling is the result of interactions between multiple biochemical mechanisms such as oxidative stress, tissue inflammation, endothelial dysfunction, and sympathetic overactivation and blood pressure overload [48,49]. The increased carotid artery IMT in patients with OSA appeared to be associated with a rise in serum inflammatory markers. In guinea pigs, although CIH caused sympathetic overactivation, it did not evoke generalized endothelial dysfunction and did not increase liver NF-kB or the aconitase to fumarase ratio, indicative of mitochondrial oxidative stress and inflammation, suggesting that it may cause less oxidative stress and inflammation than it does in rats [50]. Although we cannot rule out the possibility that CIH was eliciting oxidative stress and/or inflammation in other regions of the body, these results suggest that these effects of CIH could generally be less pronounced in the guinea pig than in rats. This may account for the lack of an increase in carotid artery IMT that we observed.

Apparently, however, pulmonary artery remodeling in this species is more sensitive to CIH, since both collagen fiber content and IMT were increased. There is evidence that CIH causes an increases in the thickness of the medial layer, as well as elastin fiber disruption, and interlaminar collagen accumulation. This induce arterial stiffness, thereby contributing to vascular resistance and arterial blood pressure elevation [5,51]. Remodeling of the adventitia, media, and intima in the pulmonary arterial bed has also been associated with increased pulmonary pressure. Several studies correlate intermittent hypoxia and pulmonary hypertension in mouse and rat models [15,52,53,54], including those from our laboratory [14]. In contrast, other studies suggest that intermittent apnea-related hypoxia is not sufficient to cause sustained PH [55,56]. We observed that 4 weeks of CIH did not cause PH in guinea pigs, possibly because the extent of remodeling that developed was insufficient to affect baseline pulmonary vascular resistance. However, we cannot exclude the possibility that a prolonged exposure to CIH may result in greater structural and functional damage to the pulmonary circulation, including an increase in PAP.

It is interesting to note that elevated levels of NO were found in CIH guinea pigs (Figure 6) when plasma NO metabolites (nitrites and nitrates) were measured as an index of NO bioavailability [57]. The NO oxidation products nitrite and nitrate are recognized as sources of recycled NO. The activation of the nitrate–nitrite–NO pathway is an alternative source of NO that may have an anti-hypertensive effect [58]. This conversion may be particularly effective under conditions of hypoxia and acidosis, such as occur in skeletal muscle during exercise. It is noteworthy that the increase in plasma NO metabolites in guinea pig was similar to that we observed using the same CIH protocol in the rat [14], suggesting that this effect occurs independently of the CB and does not require a rise in plasma angiotensin II or endothelin.

Conversely, no changes were found in the plasma levels of L-arginine or its metabolite ADMA, a recognized cardiovascular risk factor [59]. In contrast, the plasma levels of SDMA, an isomer of ADMA, were elevated (≈66%) in CIH-exposed guinea pigs with systemic arterial hypertension but not pulmonary hypertension. ADMA and SDMA result from post-translational methylation of L-arginine residues in proteins by protein arginine methyltransferases (PMRTs). Therefore, their levels increase after the proteolysis of methylated proteins [60]. ADMA acts as an endogenous competitive inhibitor of NOS [60,61], and although SDMA does not directly inhibit NOS, it competes with L-arginine for cellular uptake [60]. Elevated levels of ADMA, but not SDMA, have been implicated in endothelial dysfunction in many pathological conditions [62,63,64], and there is evidence that CIH in humans leads to an increase in plasma ADMA levels that is dependent upon dimethylarginine dimethylaminohydrolase 2 (DDH2), one of the enzymes that mediates its breakdown [65]. Further studies are needed to investigate a possible correlation between SDMA levels and CIH exposure effects.

One of the hallmark effects of CIH in rodents [66] is a rise in the plasma endothelin concentration. There is evidence that this, as well as an increased expression of ET_A_ receptors, contributes to systemic vascular constriction and a rise in MABP in rats and to the hypersensitivity of the CB evoked by CIH [44,67]. Endothelin has also been implicated in the CIH-induced vascular remodeling of the aorta and mesenteric arteries in mice [19,68] and in pulmonary vascular remodeling associated with the hypoxia/Sugen model of pulmonary hypertension in rats [69]. In humans, however, although OSA has been shown to increase plasma endothelin levels [70], it is unclear whether this is a direct effect or is due to co-morbidities associated with this condition [71]. Also, a role for endothelin in chronic OSA-associated hypertension appears not to have been confirmed and endothelin has been shown to not contribute to the hemodynamic effects of acute intermittent hypoxia in humans [72]. We observed that CIH had no effect on plasma ET-1, suggesting that a degree of vascular remodeling of the carotid and pulmonary arteries can occur independently of an increase in ET-1. However, in view of the finding by Gras et al. [19] that the increase in the aortic IMT in mice was prevented by the ET_A/B_ receptor antagonist bosentan, it is possible that the lack of an effect of CIH on the IMT in the aorta and carotid arteries may have been due to the absence of a rise in ET-1 in the guinea pig.

CIH in animal models has also been shown to increase plasma angiotensin II levels [27]. Increased circulating angiotensin II levels contribute to the rise in MABP induced by CIH [73], in part by acting on CNS blood-pressure-regulating centers to increase sympathetic outflow [74,75,76]. Angiotensin II has also been shown to promote the CB hypersensitivity [77], oxidative stress [78,79], and pulmonary [80] and aortic vascular remodeling [81] associated with CIH/OSA. In guinea pigs, however, we found that CIH did not affect the plasma levels of angiotensin II, suggesting that the rise in MABP it evokes in this species does not depend on an increase in its circulating concentration. In addition, it appears that the increase in IMT and collagen content we observed did not require an increase in circulating angiotensin II levels, although we cannot exclude the possibility that local activation of the renin–angiotensin–aldosterone system within the lung could have occurred and contributed to pulmonary artery remodeling.

Finally, the findings on the plasma levels of catecholamines (CAs) deserve a few remarks. In CIH guinea pigs, there was a large increase in the plasma levels of the CAs NE and E. Since sympathetic nerve endings are the origin of plasma NE, whereas E is secreted from the adrenal medulla (AM), the plasma levels of NE and E represent an index of general sympathetic tone [82,83]. Interestingly, these results, together with increases in MABP (10 mmHg) and HR (25% increase), point to an activation of the sympathetic system after CIH in the guinea pig. Given that CIH-induced hypertension and increased sympathetic activity may be mediated by CB overactivity, as it is prevented by chronic bilateral sectioning of the carotid sinus nerve [10], these responses represent a paradoxical result in an animal lacking CB activity and carotid sinus nerve afferent activity [32,84] and a blunted ventilatory response to the full range of hypoxia. These respiratory responses are typical of mammals and humans adapted to high altitude [85]. Furthermore, the absence of hypoventilation during a Dejours test [16] indicates that, unlike in rats and other species, the CB does not contribute to normoxic ventilation in guinea pigs. Conversely, the ventilatory response to hypercapnia is similar before and after CIH treatment and is comparable to that observed in the rat [32], demonstrating that central chemoreceptors respond normally to hypercapnic stimuli in guinea pigs, as reported by Schwenke et al. [84]. Taken together with the sympathetic activation following CIH exposure in guinea pigs, our results demonstrate that other oxygen sensors integrated into the respiratory and sympathetic networks of the brainstem could monitor tissue oxygen in the CNS independently of CB activity and elicit respiratory and/or sympathetic excitatory responses during central hypoxia [86].

In conclusion, the present study demonstrates that 30 days of the exposure of guinea pigs to CIH (FiO_2_, 5% for 40 s, 30 cycles/h) induces pulmonary artery remodeling but does not alter endothelial function or the contractile response to PE in these arteries. On the other hand, this model was associated with increased MABP (20%, *p* < 0.05) and altered responses to PE and carbachol in the aorta without changes in its wall structure. Since these alterations occurred in the functional absence of a CB influence, we conclude that they are independent of CB sensitization. Thus, it appears that there must be other oxygen sensors with the capacity to alter the autonomic control of the heart in CIH as well as vascular structure and function. Whether this finding can be extended to OSA patients and those living and adapted to high altitude remains to be investigated.

## 4. Materials and Methods

This investigation conformed to the Guide for Care and Use of Laboratory Animals published by the European Union Directive for Protection of Vertebrates Used for Experimental and Other Scientific Ends (2010/63/EU). Protocols were reviewed and approved by the University of Valladolid Institutional Ethical Committee for Animal Care and Use (Project Approval Ethical Code: 4505502)

### 4.1. Chronic Intermittent Hypoxia Protocol

Experiments were conducted on 24 male 3-month-old Hartley guinea pigs from Harlam Iberica housed (3/cage) in an ad hoc room of the vivarium with a light–dark cycle of 12 h at a room temperature of 20–23 °C. Animals were provided standard chow and water supplemented with vitamin C (Farma Bayer, Spain; 1 g/L) ad libitum. After one week of adaptation, animals were randomly distributed into two groups of 12. One group was maintained in a normoxic atmosphere (control animals, C) and the other group was exposed to an intermittently hypoxic intermittent atmosphere (CIH animals; 21% O_2_–80s/5% O_2_–40 s; 8 h/day) as previously described [15]. Briefly, guinea pigs were placed in Plexiglas chambers. A timed solenoid valve system was used to distribute nitrogen and air to each chamber. CIH guinea pig chambers (*n* = 12) were flushed with air–nitrogen for 20 s to achieve hypoxia (5% O_2_), followed by 80 s of compressed air to restore normoxia (21% O_2_) before starting the next hypoxia cycle. Cycles for both groups were repeated 8 h/day for 30 days. The level of FiO_2_ in the chambers was controlled throughout the hypoxia protocol using an O_2_ analyzer (Oxydig, DRÄGER, Lübeck, Germany). Body weight was measured weekly.

### 4.2. In Vivo Pulmonary and Systemic Blood Pressure Measurements

For systemic arterial pressure recordings, anesthetized guinea pigs (ketamine 100 mg/Kg + diazepam 2 mg/Kg; i.p.) were tracheostomized and pump-ventilated with room air (CL Palmer, UK; 60 cycles/min and positive expiratory pressure of 2 cm H_2_O) as previously described (15). The right carotid artery was cannulated and connected to a physiological pressure transducer (AD Instruments, Oxford, UK). Animals were allowed to stabilize for 20 min following surgery and then the baseline heart rate (HR) and arterial blood pressure (ABP) was continuously monitored. Signals were recorded and stored (BIOPAC Systems, Inc. MP 150, Goleta, CA, USA; Acknowledge 3.9.1) for subsequent analysis (heart rate, systolic and diastolic pressure, pulse pressure).

In order to simultaneously measure PAP, in open-chest animals the main PA was cannulated through the right ventricle (RV) and connected to a pressure transducer (Transpac IV; ICU Medical, Inc., San Clemente, CA 92673, USA). Systolic and diastolic pressure measurements were performed under normoxic and hypoxic conditions (4 min at FIO_2_ = 0.10). To record the effect of hypoxia on PAP, the inlet of the respirator was connected to a balloon filled with a mixture of 10% O_2/_90% N_2_. The recovery profile was evaluated by pumping in air with an FIO_2_ = 0.21. Arterial oxygen tension (PaO_2_) was analyzed in blood sampled from the carotid artery immediately after hemodynamic measurements. At the end of experiments, animals were euthanized by the administration of a lethal dose of sodium pentobarbital.

### 4.3. Vessel Reactivity and Endothelial Function

Studies of tension development were performed on rings from aortas and pulmonary and carotid arteries. Following euthanasia, the thoracic aorta and the left carotid artery were excised and transferred to dishes filled with Krebs bicarbonate buffer solution (NaCl 118 mM, KCl 4 mM, MgSO_4_ 1 mM, CaCl_2_ 1.8 mM, KH_2_PO_4_ 1.0 mM, NaHCO_3_ 24 mM, glucose 5 mM) and cleared of periadventitial tissue under a dissection microscope. Lungs were extracted and the PA isolated and cleaned of fat and connective tissue. Arteries were cut into rings 3 mm long and mounted on a conventional wire myograph (DMT) in Krebs solution maintained at 37 °C and bubbled with 21% O_2_, 5% CO_2_, and remainder N_2_. Care was taken not to touch the inner surface of the blood vessels.

After a period of stabilization, the arteries were contracted using 80 mM KCl to determine their viability and contractile capacity (reference contraction). After a second stabilization period, the vascular rings were contracted with cumulative concentrations of PEPE (0.1 µM–30 µM). Contractile responses were expressed as a percentage of the contraction induced by 80 mM KCl. Endothelial function was then assessed by testing the relaxant effect of cumulative doses of carbachol (10 nM–0.1 mM) on rings pre-contracted with 30 µM PE. Relaxant responses to carbachol were expressed as a percentage of PE-induced tone. The failure of carbachol to elicit the relaxation of vessel rings previously incubated with PE was taken as proof of endothelial damage. Endothelium-dependent contractile and relaxation function were assessed by testing the L-NAME (0.1 mM) effect on the dose response contractions to PE (0.1 µM–30 µM) and in on the relaxant effects of carbachol, respectively.

### 4.4. Fulton Index

To measure possible RV hypertrophy, immediately after death the heart was removed and the RV and the left ventricle plus the septum (LV + S) were dissected out and weighed separately, and the ratio between the RV and LV + S weights (RV/LV + S) was calculated as the Fulton index.

### 4.5. Morphometric and Histological Assessments

To assess vessel wall remodeling, aorta, carotid, and PA rings were collected at the end of the in vivo analysis. They were fixed in 10% paraformaldehyde for 48 h at room temperature and stored in PBS at 4 °C. For processing, they were embedded in paraffin, sectioned at 5 μm, and stained with Masson’s trichrome for histological examination. After staining, the sections were visualized with a NIKON Eclipse 90i Fluorescence Microscope and photographed with a NIKON model DS-Fi3 CMOS camera (5.9 mP). Images were taken in from serial sections of each tissue at ×4, ×10, and ×20 magnification.

Arterial intima–media thickness, lumen area, and collagen (expressed as a percentage of total IMT area) were measured in five tissue sections per animal using Image J/Fiji software. To quantify the percentage of collagen per vessel area, all photographs were first visualized for standardization using the color threshold. The same threshold was then applied to all the images.

### 4.6. L-arginine and Metabolites

For the simultaneous analysis of the several endogenous substances involved in the NO-generating pathway, the plasma levels of L-arginine, asymmetrical dimethyl-L-arginine (ADMA), and symmetrical dimethyl-L-arginine (SDMA) were measured by a two-step HPLC-FD with fluorescence detection. Plasma samples were prepared by adding 1 mM stock solution of the internal standard monomethyl-arginine (MMA) prepared in 10 mM HCl and using a pre-conditioned solid extraction cartridge (Oasis MCX cation-exchange SPE columns (30 mg, 1 cc) supplied by Waters as previously described [16].

Briefly, a 0.2 mL sample was mixed with 0.1 mL of internal standard (MMA) and 0.7 mL of PBS. The Oasis MCX SPE columns were preconditioned with 1 mL of ammonia/water/methanol (10/40/50) and 1 mL of water. After sample addition, the columns were consecutively washed with 1 mL of 100 mM HCl and 1 mL of methanol. Analytes were eluted with 1 mL of ammonia/water/methanol (10/40/50). All washing and elution steps were performed by vacuum suction. Column eluates were evaporated under a vacuum lyophilizer and frozen at −20 °C until injection into the HPLC-FD. Before injection, the amino acids were derivatized with 0.1 mL of o-phthaldialdehyde (OPA) reagent containing 3-mercaptopropionic acid added to the residue. The derivatized amino acids were separated by isocratic reversed-phase chromatography performed on a C18 column (Strata-XL-C 100 µm, 30 mg/mL Phenomenex) using a mobile phase consisting of potassium phosphate buffer (50 mmol/L, pH 6.5) containing 8.7% acetonitrile at a flow rate of 0.3 mL/min. Fluorescence detection was performed at excitation and emission wavelengths of 340 and 455 nm, respectively. Peaks were quantified based on peak area.

### 4.7. Plasma Chemistry of Vasoactive Agents

A method based on the Griess reaction was used to measure the nitrites and nitrates in plasma, as described in [14,87]. Briefly, plasma samples were incubated with nitrate reductase (Sigma-Aldrich, Dorset UK) for 2 h at room temperature; then, Griess reagent (Sigma-Aldrich, Dorset, UK) was added to the sample. The absorbance was read at 540 nm with a microplate reader 10 min later and interpolated to a standard curve with different concentrations of sodium nitrate (0–60 µM).

Endothelin-1 (ET-1) and angiotensin II (ANG II) levels were determined via ELISA as previously described [50].

The plasma levels of norepinephrine (NE) and epinephrine (E) were measured using high-pressure liquid chromatography (HPLC) as previously described [14].

The activity of aconitase/fumarase was determined as previously described [50]. Briefly, the liver was homogenized on ice in TES buffer (10 mM Tris, pH 7.4, 250 mM sucrose and 1 mM EDTA; 10 mL/g of tissue) and the mitochondria were isolated and centrifuged at 4 °C [50]. The resulting pellet was resuspended in 0.5 mL of TES buffer containing 0.2% TritonX-100 to lyse the mitochondria.

The enzymatic reaction was measured for 3 min in 30 mM sodium isocitrate, 50 mM sodium isocitrate, 50 mM Tris-HCl, pH 7.4, and 0.6 mM MnCl_2_ for the determination of aconitase activity and 50 mM sodium malate, 50 mM sodium phosphate buffer, and pH 7.4 for the fumarase activity. Enzyme activities were measured spectrophotometrically according to the increase in optical density at 240 nm and expressed as the activity ratio.

NF-ĸB measurement was performed in liver nuclear extracts (Active Motif kit, Waterloo, Belgium) using a p65 TransAM transcription factor assay kit (40096; Active Motif, Waterloo, Belgium). NF-kB activation was detected by incubation of the primary anti-NF-kB antibody with the nuclear samples. The HRP-conjugated anti-IgG secondary antibody allows its detection by spectrophotometry by measuring the absorbance of the samples at 450 nm.

### 4.8. Drugs

The drugs used were: carbachol, PE, and L-NAME (all from Sigma-Aldrich; Madrid, Spain). Drugs were kept at −20 °C and freshly dissolved in distilled water to the appropriate concentration expressed as final molar concentration.

### 4.9. Data Presentation and Statistics

Data were evaluated using Graph Pad Prism Software version 6 (GraphPad Software Inc., San Diego, CA, USA) and presented as mean ± standard error of the mean (S.E.M.).

Mean value comparisons were performed using an unpaired Student’s *t*-test and by two-way ANOVA with Sidak’s multi-comparison test according to the structure of data. Differences were considered statistically significant at a *p*-value < 0.05.

## Figures and Tables

**Table 1:** Body weight, erythrocytes, hematocrit, arterial blood PO_2_ and PCO_2_, and erythropoietin (EPO) in control (C) and chronic intermittent hypoxia (CIH) guinea pigs. Data are expressed as mean ± standard error (SEM) from N = 8 C and N= 8 CIH animals. (* *p* < 0.05; ** *p* < 0.01).

	C	CIH
Body weight (g)	791 ± 19	715 ± 14 **
Hematocrit (%)	41.5 ± 0.8	41.4 ± 0.8
EPO (mU/mL)	141 ± 17	214 ± 52 *
pO_2_ (mm Hg)	65 ± 6	64 ± 6
pCO_2_ (mm Hg)	36 ± 2	38 ± 3

**Table 2:** Vascular responses to 80 mM KCl in the aorta, carotid, and pulmonary artery in control (C) and chronic intermittent hypoxia (CIH) guinea pigs; “n” represents the number of arteries from N = 8 animals/group.

	Aorta		Carotid Artery		Pulmonary Artery	
	C	CIH	C	CIH	C	CIH
Mean (mN)	9.7 ± 1.4	6.5 ± 1.8	16.0 ± 1.7	16.9 ± 2.4	10.0 ± 1.4	10.3 ± 0.7
n	15	8	19	16	15	12

**Table 3:** Endothelin-1 (ET-1), Angotensin II (ANG II), Atrial natriuretic peptide (ANP), Vascular endothelial growth factor (VEGF), and the Catecholamines norepinephrine (NE) and epinephrine (E) contents in plasma in control and CIH guinea pigs. Data are expressed as mean ± standard error (SEM) N = 8 animals. Unpaired *t*-test (* *p* < 0.05).

Guinea Pig	C	CIH
ET-1 (pg/mL)	19.9 ± 1.9	21.5 ± 1.8
ANG II (pg/mL)	355 ± 49	412 ± 67
ANP (pg/mL)	234 ± 15	209 ± 6
VEGF (pg/mL)	2.5 ± 0.2	2.6 ± 0.2
NE (pmol/mL)	7.2 ± 1.7	80.7 ± 24.0 *
E (pmol/mL)	11 ± 0.4	64 ± 20 *

## Data Availability

The data that support the findings of this study are available from the corresponding author upon request. The data are pending upload to the repository of the University of Valladolid (UVaDoc).
